# Schwannoma of the Lower Limb: A Case Report

**DOI:** 10.7759/cureus.66616

**Published:** 2024-08-11

**Authors:** Nikolozi Kutalia, Magda Bolkvadze, Mehmet N Erdem

**Affiliations:** 1 Medicine, David Tvildiani Medical University, Tbilisi, GEO; 2 Internal Medicine, Tbilisi State University, Tbilisi, GEO; 3 Orthopedics and Traumatology, Hisar Intercontinental Hospital, Istanbul, TUR; 4 Orthopedics and Traumatology, Isik University, Istanbul, TUR

**Keywords:** cerebellopontine angle tumours, antoni a and antoni b areas, painless nodule, benign peripheral nerve sheath tumor, peripheral schwannoma

## Abstract

Schwannoma is a benign tumor of the peripheral nerve sheath and is a unique clinical entity when localized to a lower limb. Growing as a painless nodule, it might be misdiagnosed by many medical professionals as another benign soft tissue skin condition, such as lipoma, myxoma, or ganglion cyst. Definitive diagnosis of peripheral schwannoma is made by biopsy and histopathologic evaluation, followed by surgical excision, which is the definitive treatment of the tumor. Classic symptoms of schwannoma of the lower limb are peripheral neuropathy (tingling, burning sensations) and motor impairment (weakness, paralysis of the affected limb). MRI imaging and biopsy are the most useful diagnostic methods for peripheral schwannoma, followed by surgical excision, which is the treatment of choice. Postoperative complications, if present, are minimal and rare. Because of the slow-growing nature of the tumor and the complexity of the lower limb's nervous and structural network, it is often asymptomatic and is challenging to diagnose at a primary stage. That is why we want to spread awareness and draw the reader's attention to this rare case of a patient with schwannoma on the left lower limb.

## Introduction

Arising from Schwann cells of the peripheral nerve sheath, schwannomas comprise one of the rarest benign soft tissue tumors [1]. They are predominantly localized to the head and neck region, most commonly presenting at the cerebellopontine angle as an acoustic neuroma of CN VIII [2]. However, they may invade other cranial and peripheral nerves. Their occurrence in lower limbs is extremely rare, comprising only 1% of schwannoma cases [3,4], and when present, they often mimic a ganglion cyst, Morton's neuroma, lipoma, or vascular malformation [5]. For this reason, they pose a challenge for many doctors and are often difficult to diagnose at the initial stage. In this case report, we want to present a case of a 22-year-old female diagnosed with schwannoma of the left lower limb, as well as discuss diagnostic approaches and management strategies in this specific anatomical context.

## Case presentation

A 22-year-old female patient from Tbilisi, Georgia, presented with a protruding, palpable, tender, swollen mass on her left lower leg, which had been noticeable for three months. The patient exhibited normal vital signs during the examination and was fully oriented, with no cognitive deficits. The patient had no family history of neurofibromatosis or other neurocutaneous disorders. Initially, differential diagnoses by the doctors included ganglion cyst, lipoma, or myxoma; however, diagnostic studies revealed abnormalities inconsistent with the typical presentation of these conditions.

**Figure 1:**
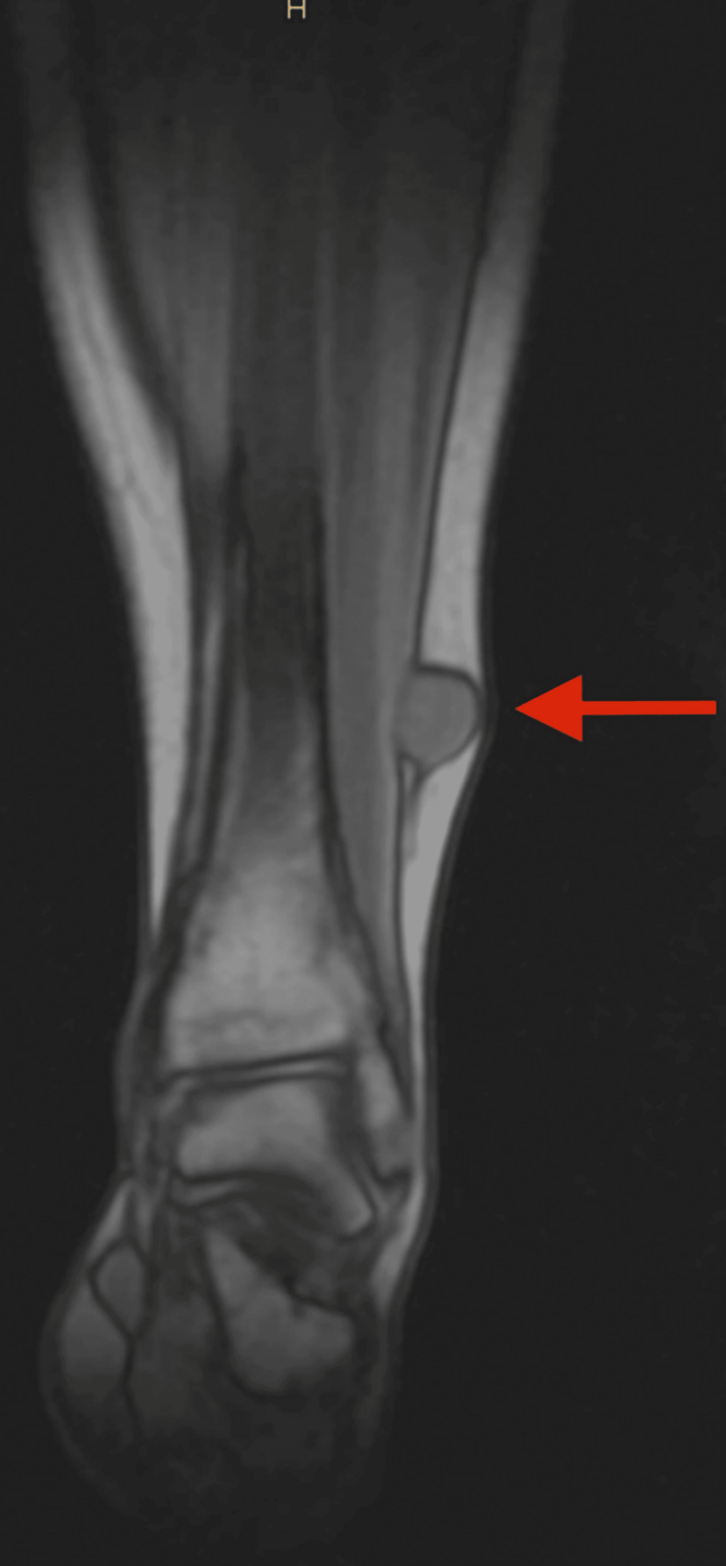
Pre-operative MRI of the left tibia showing a well-defined, oval-shaped mass, measuring 16x22mm (arrow).

MRI of the left tibia with contrast enhancement, employing sagittal and transverse slices, revealed a sizable mass measuring 16x22mm on the ventral surface of the lower third of the left tibia within the lateral subcutaneous fat tissue (Figure 1). The mass exhibited distinct, well-defined contours and a homogenous structure. Notably, it exerted compression on the extensor digitorum muscle without infiltrating or invading it or any surrounding muscles or tissues. The enhancement of the image after administration of the contrast highlighted the neoplasm, which was indicative of schwannoma; however, a biopsy was deemed necessary for a definitive diagnosis.

**Figure 2:**
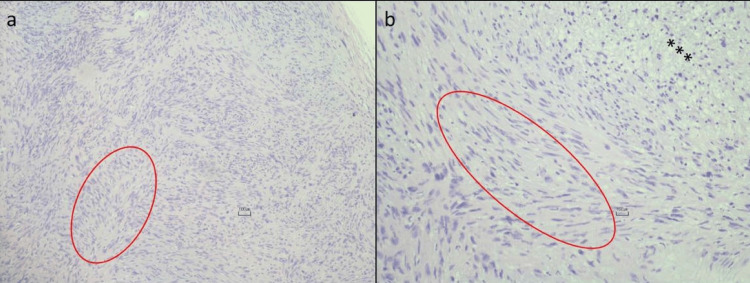
Histopathologic findings showing spindle cells distributing nuclear palisading pattern (circle), hypercellular (Antoni A) (circle), and myxoid, hypocellular areas within the tumor (Antoni B) (stars).

The biopsy, which was performed in Istanbul, Turkey, showed a tumor composed of spindle cells with narrow, elongated nuclei without any pleomorphism or increased mitotic activity (Figures 2a, 2b).

**Figure 3:**
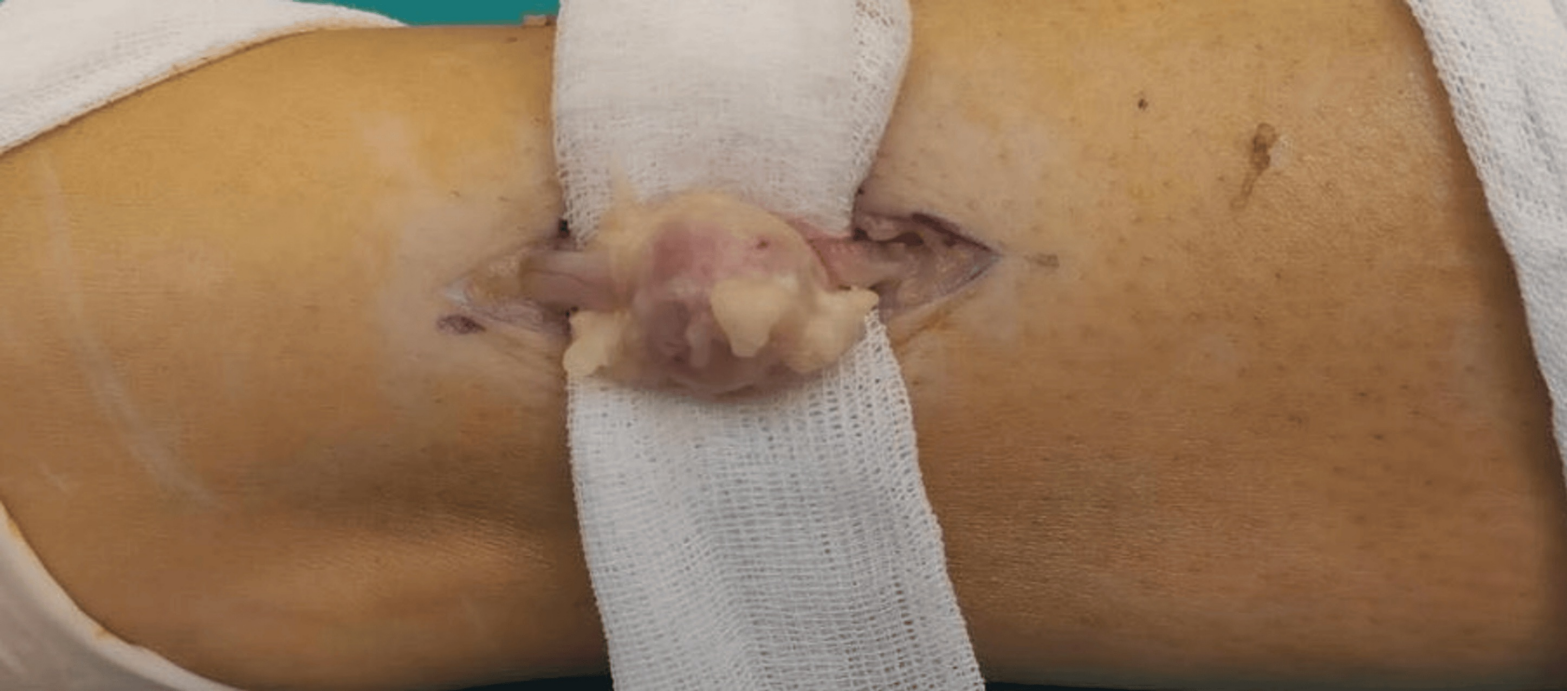
Intraoperative schwannoma showing distinct, well-circumscribed, encapsulated mass.

**Figure 4:**
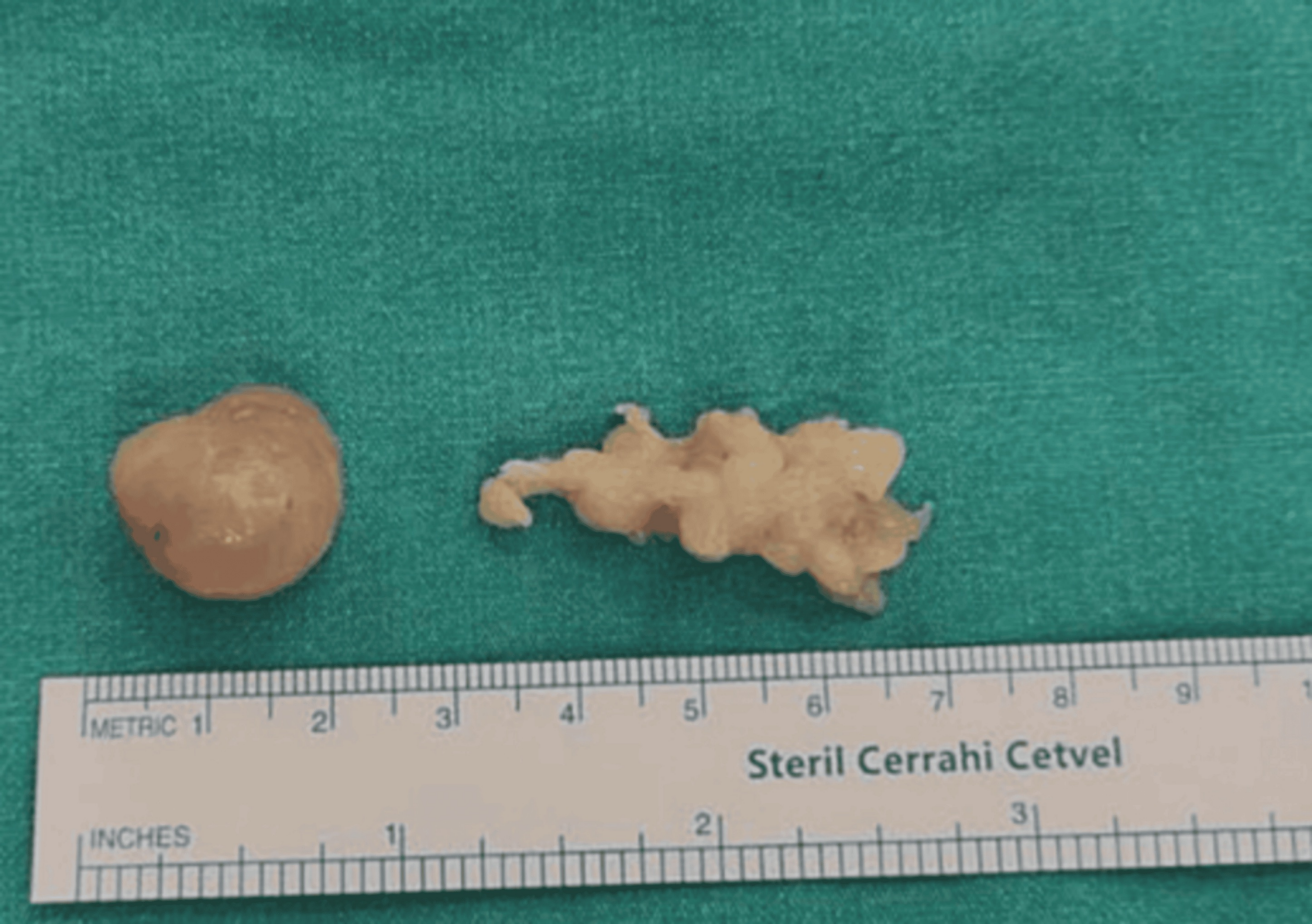
Schwannoma after excision showing a well-defined, oval-shaped tumor with a smooth surface.

As the diagnosis has been confirmed after biopsy, the decision to perform invasive surgical intervention for removing the mass was validated. The patient underwent an invasive operation under local anesthesia, during which schwannoma was successfully excised (Figures 3, 4).

Subsequently, the patient was discharged from the hospital shortly after the procedure. In the ensuing three months, the patient felt tingling and numbness and also had difficulty walking due to weakness in the left lower leg. However, the patient was reassured by the fact that these symptoms were expected and temporary. On a one-year follow-up, the patient was asymptomatic, no longer had tingling, numbness, or weakness, and exhibited no notable complications, further confirming the favorable postoperative outcome and prognosis of the tumor.

## Discussion

Schwannomas are tumors derived from Schwann cells, responsible for myelination of the peripheral nerve fibers and, when present in the form of bilateral acoustic neuroma, are closely associated with a neurocutaneous tumor - neurofibromatosis type 2 [6]. They are typically encapsulated, well-defined tumors with biphasic patterns under the microscope and consist of two components: Antoni A areas, which are densely packed with spindle-shaped cells forming palisades or Verocay bodies, and Antoni B areas, which are more loosely arranged with hypocellular myxoid stroma [7]. Immunohistochemistry shows expression of S-100 protein.

They most commonly arise in the distribution of the eighth cranial nerve in the head and neck region. Hence, their presence on the lower limb is exceptionally rare. In this anatomical context, it most commonly presents as a growing, painless mass on the lower extremity. Peripheral schwannomas are mostly asymptomatic due to their slow-growing nature; however, due to compression of adjacent nervous structures, they may also cause painless edema, peripheral neurologic symptoms (burning, tingling sensations), and motor paralysis [8].

MRI is crucial in assessing the tumor's size, shape, borders, and surrounding edema in case of existence, helps understand if the surrounding structures are invaded, and aids in making an accurate diagnosis based on changing the signal intensity [8]. Schwannomas typically appear as a well-circumscribed, encapsulated mass, most commonly up to 5cm, and enhanced with contrast administration [9]. After confirmation of the diagnosis with imaging and histopathologic diagnostic methods, the preferred choice of treatment for schwannomas arising in the extremities is complete surgical excision, even if the tumor has not spread out of the capsule [10]. Postoperative outcome and prognosis of the tumor are overall good, aside from minor residual weakness and neurologic symptoms, which disappear over time. The rate of recurrence or malignant transformation of the tumor is low as well [11].

## Conclusions

Schwannoma of the lower limb comprises 1% of all schwannoma cases, making it exceedingly rare. This may cause initial diagnostic ambiguity; however, advanced imaging and histopathologic evaluation aid in the precise identification of schwannoma and the planning of optimal management strategies. Despite minor postoperative complications, the prognosis of peripheral schwannoma is favorable after surgical excision since it is a benign, localized tumor.
